# Myostatin as a mediator of sarcopenia versus homeostatic regulator of muscle mass: insights using a new mass spectrometry-based assay

**DOI:** 10.1186/s13395-015-0047-5

**Published:** 2015-07-15

**Authors:** H. Robert Bergen, Joshua N. Farr, Patrick M. Vanderboom, Elizabeth J. Atkinson, Thomas A. White, Ravinder J. Singh, Sundeep Khosla, Nathan K. LeBrasseur

**Affiliations:** Department of Biochemistry and Molecular Biology, Mayo Clinic College of Medicine, Rochester, MN 55905 USA; Medical Genome Facility-Proteomics Core, Mayo Clinic College of Medicine, Rochester, MN 55905 USA; Division of Endocrinology, Department of Medicine, Mayo Clinic College of Medicine, Rochester, MN 55905 USA; Robert and Arlene Kogod Center on Aging, Mayo Clinic College of Medicine, Rochester, MN 55905 USA; Division of Biomedical Statistics and Informatics, Department of Health Sciences Research, Mayo Clinic College of Medicine, Rochester, MN 55905 USA; Department of Laboratory Medicine and Pathology, Mayo Clinic College of Medicine, Rochester, MN 55905 USA; Department of Physical Medicine and Rehabilitation, Mayo Clinic College of Medicine, 200 First Street SW, Rochester, MN 55905 USA

**Keywords:** Myostatin, Aging, Sarcopenia, Skeletal muscle mass, Strength, Body composition

## Abstract

**Background:**

Myostatin is a protein synthesized and secreted by skeletal muscle that negatively regulates muscle mass. The extent to which circulating myostatin levels change in the context of aging is controversial, largely due to methodological barriers.

**Methods:**

We developed a specific and sensitive liquid chromatography with tandem mass spectrometry (LC-MS/MS) assay to measure concentrations of myostatin and two of its key inhibitors, follistatin-related gene (FLRG) protein and growth and serum protein-1 (GASP-1) in 80 younger (<40 years), 80 older (>65 years), and 80 sarcopenic older women and men.

**Results:**

Older women had 34 % higher circulating concentrations of myostatin than younger women. Per unit of lean mass, both older and sarcopenic older women had >23 % higher myostatin levels than younger women. By contrast, younger men had higher myostatin concentrations than older men with and without sarcopenia. Younger men had approximately twofold higher concentrations of myostatin than younger women; however, older women and sarcopenic older women had significantly higher relative myostatin levels than the corresponding groups of men. In both sexes, sarcopenic older subjects had the highest concentrations of FLRG. Circulating concentrations of myostatin exhibited positive, but not robust, correlations with relative muscle mass in both sexes.

**Conclusions:**

Our data suggest that myostatin may contribute to the higher prevalence of sarcopenia in women but acts as a homeostatic regulator of muscle mass in men. Moreover, this new LC-MS/MS-based approach offers a means to determine the extent to which myostatin serves as a biomarker of muscle health in diverse conditions of muscle loss and deterioration.

**Electronic supplementary material:**

The online version of this article (doi:10.1186/s13395-015-0047-5) contains supplementary material, which is available to authorized users.

## Background

Over 50 years ago, circulating tissue-specific growth inhibitors were hypothesized to explain how tissue sizes are controlled [1]. Considerable work has since established growth and differentiation factor (GDF)-8, or myostatin, as a robust negative regulator of skeletal muscle mass. Myostatin is a member of the transforming growth factor β (TGF-β) superfamily that is highly enriched in skeletal muscle [2]. It is synthesized as a precursor protein and forms a disulfide-linked homodimer. The signal peptide of the myostatin precursor protein is removed by proteolytic cleavage to form promyostatin [2, 3]. Furin protein convertases within the Golgi apparatus or the extracellular space cleave promyostatin to generate N- and C-terminal fragments [4, 5], which are bound non-covalently and form a latent myostatin complex. The N-terminal is an inhibitory prodomain, referred to as propeptide, and the C-terminal represents the mature biologically active form of myostatin. The latent complex is disassembled by BMP1/tolloid proteinases [6], which enables the mature C-terminal dimer to bind to the activin type IIB receptor (ActRIIB). Through ActRIIB and its downstream effectors, particularly Smad2/3, myostatin simultaneously activates protein degradation and inhibits protein synthesis in adult skeletal muscle [7, 8]. As a result, myostatin is a promising therapeutic target for conditions of compromised muscle health, including age-related sarcopenia, disease-associated cachexia, congenital myopathies, muscular dystrophies, disuse atrophy, and trauma.

Despite strong evidence that genetic and pharmacological modulations of myostatin abundance and/or activity impact muscle mass in multiple species [9–13], data on circulating myostatin concentrations in humans are sparse and conflicting. In an early cross-sectional study, serum myostatin levels measured by radioimmunoassay (RIA) were reported to be significantly elevated with advancing age and declining lean mass, suggesting that myostatin may serve as a biomarker of sarcopenia in women and men [14]. While provocative, no subsequent study has been able to corroborate these findings. Indeed, recent studies measuring concentrations in the serum using enzyme-linked immunosorbent assays (ELISAs) have reported either no change or a decline in circulating myostatin levels in older compared to younger persons [15–17]. Of further concern, ELISA-based approaches for measuring circulating myostatin concentrations have yielded highly erratic values for healthy adults, ranging from just over 4 [15] to over 100 [18] and even 32,500 ng/ml [19].

The reasons for these inconsistent findings are not clear but likely stem from the complexities of measuring myostatin in clinical samples (reviewed in [20]). In particular, some assays have not fully appreciated the homology between myostatin and other circulating TGF-β superfamily members, which can result in cross-reactivity. For example, assays for myostatin may not exclude GDF-11, which shares 90 % homology with myostatin in its C-terminal biologically active domain [21]. Lack of specificity has likely contributed to overestimates of serum myostatin levels in several previous studies. The development and use of antibodies with higher affinity and increased specificity for myostatin have undoubtedly improved the accuracy of ELISA-based approaches [15, 17]; however, these proprietary reagents remain unavailable to the broader research community. Antibody-based methods are also limited by relatively low sensitivity. This is an additional challenge to accurately quantify circulating myostatin, which is of relatively low abundance in human serum. It is also important to recognize that the earlier RIA study was performed prior to the discovery that in circulation, myostatin forms a latent complex when bound to its inhibitory proteins, including follistatin-related gene (FLRG) protein and growth and serum protein-1 (GASP-1) [22, 23]. Only one study has provided insight into how these proteins may change in relation to age, muscle mass, and myostatin, and it was limited to men [15]. Moreover, as reviewed above, the N-terminal propeptide also inhibits myostatin activity. However, current approaches used to measure circulating myostatin concentrations are often unable to distinguish between latent and mature forms. Collectively, these methodological limitations have hindered progress in understanding how myostatin may change in humans in the context of health, aging, and disease and serve as a biomarker for skeletal muscle mass.

To overcome these challenges, we developed a multiplexed assay combining immunoaffinity purification, liquid chromatography with tandem mass spectrometry (LC-MS/MS), and selected reaction monitoring (SRM) to specifically and accurately measure tryptic peptides from both the mature and propeptide regions of circulating myostatin and two of its inhibitory proteins, FLRG and GASP-1. We then applied this novel approach to a well-characterized, population-based sample to determine the extent to which circulating concentrations of myostatin and its inhibitory proteins change within women and men across adulthood, compare between women and men, and associate with skeletal muscle mass.

## Methods

### Study subjects

Study subjects were selected from a random sample of the population of Rochester, MN, USA, using the Rochester Epidemiology Project medical records linkage system as previously described [24]. A total of 240 subjects who met the study inclusion criteria were studied, including 120 women and 120 men. Within each sex, 40 younger (20–40 years old) and 80 older (≥65 years old) subjects were included. The older women and older men were then each divided into two groups of 40 subjects based on their relative appendicular skeletal muscle mass (rASM). All 40 of the older women and 34 of the older men with comparatively low rASM were below the cut-offs recently suggested for sarcopenia, which are ≤5.67 and ≤7.23 kg/m^2^ for women and men, respectively [25]. The three groups within each sex are herein referred to as younger, older, and sarcopenic. The Mayo Clinic Institutional Review Board approved this study, and all participants provided written informed consent.

Subjects were rigorously screened for coexisting disease using clinical records, and a health history and complete list of medications were obtained during an interview. This study only included subjects without the presence of coexisting disease and excluded postmenopausal women on hormone replacement therapy but not younger women on oral contraceptives. Individuals on medications associated with altered skeletal muscle mass or function were also excluded. Menopause was defined as the absence of menses for greater than 6 months. Using this definition, all of the younger women were premenopausal, whereas all older women were postmenopausal. All data, including serum samples, were collected in the outpatient Mayo Clinical Research Unit between November of 2000 and May of 2006. Blood samples were collected from study subjects the morning following an overnight fast and were stored at −80 °C.

### Study protocol

Height was obtained (nearest millimeter) using a wall-mounted stadiometer (Mayo Section of Engineering) and weight obtained (nearest 0.1 kg) using an electronic scale (Model 5002, Tronic, Inc., White Plains, NY, USA). Body mass index (BMI, kg/m^2^) was calculated as the ratio of weight to height squared. Total body and regional measures of lean mass (kg) and fat mass (kg) were obtained from whole-body dual-energy X-ray absorptiometry (DXA) scans (Lunar Prodigy, GE Medical Systems, Madison, WI, USA), using software version 6.10.029. The coefficient of variation (CV) for whole-body lean mass (CV = 0.6 %) has been reported previously [26].

Grip strength (kg) and knee extensor strength (kg) were assessed quantitatively with dynamometers (NK Biotechnical Corp., Minneapolis, MN, USA). Estimates of caloric expenditure (kcal/d) based on habitual levels of physical activity over the preceding year were calculated using data obtained from a validated physical activity questionnaire [27] and were adjusted for body weight as described previously [26]. Each activity was assigned a published metabolic equivalent (MET [1 MET = 3.5 mL O_2_ × kg^−1^ × min^−1^]) value obtained from the compendium of physical activities [28].

### Materials

Tris(2-carboxyethyl)phosphine hydrochloride (TCEP) and iodoacetamide were purchased from Sigma (St. Louis, MO, USA). Phosphate buffered saline (PBS) was purchased from Bio-Rad (Hercules, CA, USA). Ammonium bicarbonate was purchased from J.T. Baker (Center Valley, PA, USA). 3-[(3-cholamidopropyl)dimethylammonio]-1-propanesulfonate (CHAPS), bovine serum albumin (BSA), and trifluoroacetic acid (TFA) was purchased from Thermo Fisher Scientific (Waltham, MA, USA). Zwittergent Z3-16 was purchased from CalBiochem (EMD Millipore, Billerica, MA, USA). Dynabeads® M-280 Streptavidin was purchased from Invitrogen (Carlsbad, CA, USA). Trypsin/Lys-C Mix was purchased from Promega (Madison, WI, USA). Recombinant human myostatin (cat# 4623–10) was purchased from Biovision (Milpitas, CA, USA). Recombinant human GASP-1 (cat# 2070-GS-025), recombinant human FLRG (cat# 1288-F3-025), biotinylated anti-myostatin antibody (cat# BAF788), biotinylated anti-GASP1 antibody (cat# BAF2070), and biotinylated anti-FLRG antibody (cat# BAF1288) were purchased from R&D systems (Minneapolis, MN, USA).

### Magnetic bead preparation

Biotinylated anti-myostatin, biotinylated anti-GASP-1, and biotinylated anti-FLRG antibodies (0.25 μg/μL in PBS with 0.1 % BSA) were each immobilized to Dynabeads® M-280 Streptavidin (10 mg/mL in PBS) at a ratio of 40 μL antibody to 125 μL of magnetic bead suspension. These solutions were then combined, washed three times with PBS, and reconstituted to a final volume of 625 μl with PBS.

### Sample preparation

Standards, controls, and patient samples (400 μL) were each transferred into 1.5-mL microcentrifuge tubes and diluted with 600 μL PBS containing 0.03 % CHAPS. Fifteen microliters of the immobilized antibody mixture (0.25 μg of each antibody) was added to each microcentrifuge tube containing sample or standards and incubated overnight at 4 °C with rotation. The beads were washed three times with 500 μL PBS, and the PBS was removed before adding 15 μL of 8 M urea, 15 mM TCEP in 50 mM ammonium bicarbonate. Synthetic internal standard peptides were synthesized in the Mayo Proteomics Core. Some target peptides were prepared as extended tryptic sequence containing four to five additional amino acids at both the N- and C-terminal ends to take into account digestion efficiencies as noted in Table 1. The internal standard peptide mixture (5 μM IIYGKIPAMV*VDRCGCS, 2.5 μM AGVLRADFPLSV*VRGHQAA, 12.5 μM GLPARLQVCGSD*G*ATYRDECEL, and 0.625 μM EQ*IIYGK, VSELTEE*PDSGR) in 50 mM ammonium bicarbonate (5 μL) was added and the samples incubated for 30 min at room temperature (RT). Following reduction, samples were alkylated with 20 μL of 60 mM iodoacetamide (30 mM final) and incubated in darkness for 30 min at RT. Subsequently, the urea was diluted to 1.1 M urea with the addition of 70 μL of 50 mM ammonium bicarbonate. Finally, 5 μL of 0.2 μg/μL Trypsin/Lys-C Mix was added directly to digest all bead bound protein after incubation at 37 °C for 4 h. The digestion was terminated by adding 5 μL of 4.7 % TFA with 0.02 % Zwittergent® 3-16, and the digest was removed to an autosampler vial after separation from the beads prior to LC-MS/MS analysis.

**Table 1:** Peptides, transitions, and instrument parameters for myostatin, propeptide, GASP-1, and FLRG

	Parent mass	Product mass	Collision energy	S-lens
Myostatin				
***IPAMVVDR***	450.68	619.33	19	123
	*450.68*	*690.40*	*17*	*123*
	450.68	787.52	17	123
IIYGKIPAMV*VDRCGCS	453.65	619.44	19	123
	*453.65*	*690.50*	*18*	*123*
	453.65	763.65	16	123
EQIIYGK	425.66	480.31	13	86
	*425.66*	*593.47*	*14*	*86*
EQ*IIYGK	429.14	480.35	13	86
	*429.14*	*600.43*	*14*	*86*
Propeptide				
***TVLQNWLK***	501.18	560.32	18	131
	*501.18*	*688.60*	*18*	*131*
	501.18	801.78	18	131
SIDVKTVLQNW*LKQPESN	504.75	567.25	19	131
	*504.75*	*695.42*	*17*	*131*
	504.75	808.65	15	131
ELIDQYDVQR	639.70	680.42	21	132
	*639.70*	*808.50*	*20*	*132*
	639.70	923.77	20	132
ELIDQYD*VQR	642.92	686.48	20	132
	*642.92*	*814.36*	*22*	*132*
	642.92	929.43	21	132
GASP-1				
***ADFPLSVVR***	*502.28*	*670.53*	*18*	*131*
AGVLRADFPLSV*VRGHQAA	505.23	579.46	19	131
	*505.23*	*676.64*	*16*	*131*
VSELTEEPDSGR	659.81	789.36	20	168
	*659.81*	*890.51*	*21*	*168*
VSELTEE*PDSGR	662.73	795.31	21	168
	*662.73*	*896.60*	*21*	*168*
FLRG				
***LQVCGSDGATYR***	663.78	826.30	23	168
	*663.78*	*986.34*	*21*	*168*
	663.78	1085.46	21	168
GLPARLQVCGSD*G*ATYRDECEL	666.78	832.56	24	168
	*666.78*	*992.69*	*23*	*168*
	666.78	1092.15	22	168

Target peptides for each protein, parent and product ion masses as well as MS parameters and transitions used quantitation of each protein are indicated in bold and/or italics. Labeled amino acids (^13^C_5_^15^N-V, ^13^C_6_^15^N-L, ^13^C_5_^15^N-P_,_^13^C_2_^15^N-G, and ^13^C_3_-A) are indicated by the asterisk

### Liquid chromatography-tandem mass spectrometry

LC-MS/MS was performed using a nanoAcquity UPLC system (Waters Corporation, Milford, MA, USA) plumbed with a vented tee and coupled to a TSQ Vantage triple quadrupole mass spectrometer (ThermoScientific, San Jose, CA, USA) with an ADVANCE Captive Spray source (Michrom Bioresources). Briefly, 10 μl of digested sample was loaded onto a 0.25 μL OPTI-PAK® trap cartridge (Optimize Technologies, Oregon City, OR, USA) packed with Michrom Magic C8 (5 μm, 200 A) using 0.1 % formic acid in 2 % ACN at a flow rate of 10 μL/min for 4 min. Following loading, the peptides were eluted onto the analytical column (Michrom, Magic C18 AQ 200 A, 0.1 × 150 mm, 3 μm) with a 20-min gradient from 98 % A (0.1 % formic acid in 2 % ACN) to 40 % B (0.1 % formic acid in 80 % ACN and 10 % IPA) at 1 μL/min. The gradient was taken to 95 % B in 3 min and held for 2 min at 95 % to wash the column. The column was then equilibrated by returning the gradient to 98 % A in 3 min where it was held for 7 min for a total run time of 35 min.

LC-MS/MS was performed in positive ion mode using a spray voltage of 1400 V and a capillary temperature of 175 °C. All tryptic peptides and their corresponding labeled peptides were monitored in the (M + 2H^+^)^2+^ charge state. Transitions (Additional file 1: Table S1) were monitored in three time segments. Resolution was set at 0.7 FWHM for both Q1 and Q3. The scan time was 20 ms, and the scan width was set at 0.05 Da.

### Raw data analysis

Xcalibur Quan Browser version 2.2 was used for data processing. Peak integration was performed with the following parameters: peak detection algorithm, genesis; smoothing, 9; signal to noise threshold, 0.5. Peak areas for each SRM transition were recorded individually, and one transition per peptide was chosen for quantitation (Additional file 1: Table S1). Peak area ratios were established for each surrogate peptide and its corresponding internal standard peptide, and calibration curves were generated by plotting these ratios against protein concentration (nM). Each peptide was fit with a linear calibration curve using 1/X weighting, with the exception of VSELTEEPDSGR for GASP-1, which was fit with a quadratic curve using 1/X weighting.

### Other biochemical measures

Total 25-hydroxyvitamin D [25-(OH)D] (inter-assay CV = 7 %) was measured in serum using LC-MS/MS (API 5000; Applied Biosystems-MDS Sciex, Foster City, CA, USA). Total insulin-like growth factor 1 (IGF-1) and IGF-2 (inter-assay CVs = 6 % for both) were measured in serum by a two-site immunoradiometric assay (IRMA), after separation from their binding proteins with a simple organic solvent extraction (Diagnostic Systems Laboratories, Webster, TX, USA). Serum insulin-like growth factor binding protein 2 (IGFBP-2) (inter-assay CV = 16 %) was measured by a double antibody RIA (Diagnostic Systems Laboratories, Webster, TX, USA), whereas IGFBP-3 (inter-assay CV = 14 %) was measured in serum by a two-site IRMA (Diagnostic Systems Laboratories, Webster, TX, USA). Sex hormone-binding globulin (SHBG) (inter-assay CV = 7 %) was measured in serum by RIA (Wien Laboratories, Succasunna, NJ, USA) [29]. As described previously [30], serum sex steroids, including estrone (E_1_, inter-assay CV = 8 %), estradiol (E_2_, inter-assay CV = 8 %), and testosterone (T, inter-assay CV = 6 %) were measured using LC-MS/MS (API 5000; Applied Biosystems-MDS Sciex, Foster City, CA, USA), which allows detection of values as low as 1.25 pg/mL for both E_1_ and E_2_ and 1 ng/dL for T. The non-SHBG bound (i.e., biologically active fraction) of E_2_ and T (inter-assay CVs = 12 % for both) were measured and then multiplied by the total E_2_ or T measured by mass spectrometry to obtain the respective bioavailable E_2_ and T fractions.

### Statistical analysis

In women and men, separately, comparisons among the younger, older, and sarcopenic groups were made using an analysis of variance model. Comparisons between younger, older and sarcopenic women, and the corresponding group of men were made using an unpaired *t* test. The Mann-Whitney *U* test was used when variables were not normally distributed, as appropriate. Associations of myostatin levels with body composition, muscle strength, physical activity, and other biochemical parameters were examined using age-adjusted Spearman correlations. Testing was performed at a significance level of *P* < 0.05 (two-tailed). Analyses were performed using JMP 10.0 and SAS 9.3. Box plots (25–75 percentile), and whiskers (Tukey method) were created using GraphPad Prism 5.03.

## Results

### Validation of a LC-MS/MS assay for myostatin, GASP-1, and FLRG

**Fig. 1:**
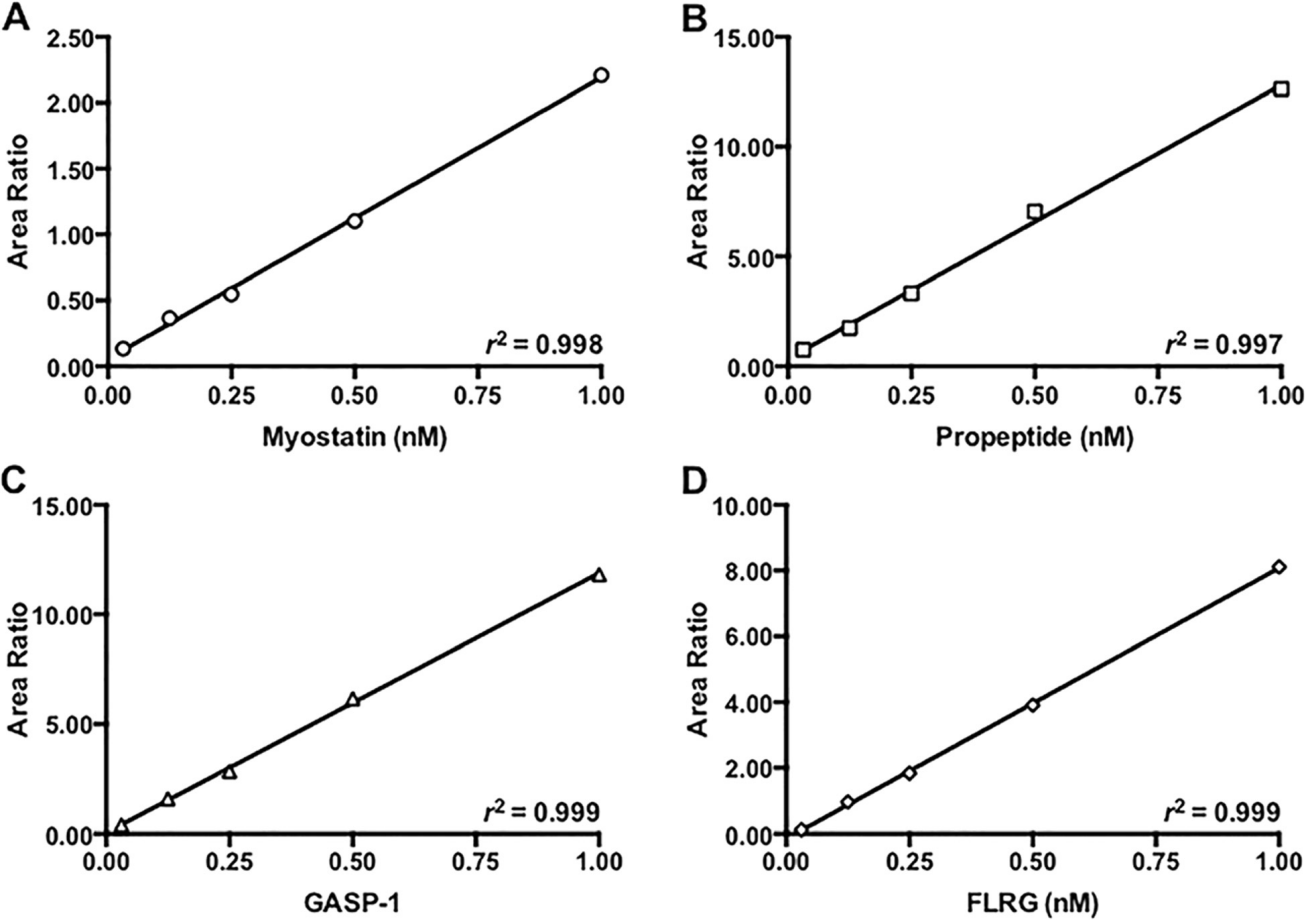
Calibration curves for LC-MS/MS measures of myostatin, propeptide, GASP-1, and FLRG. Linear calibration curves for recombinant intact myostatin (**a**), propeptide (**b**), GASP-1 (**c**), and FLRG (**d**) diluted into bovine serum albumin over a concentration range of 0.031 to 1.00 nM

The peptides, transitions, and instrument parameters for myostatin, propeptide, GASP-1, and FLRG are detailed in Table 1. The lower limits of detection (LOD, average blank concentration plus 3 SDs) for myostatin, propeptide, GASP-1, and FLRG were 0.01 (0.248), 0.015 (0.42), 0.01 (0.607), and 0.02 nM (0.258 ng), respectively. The lower limits of quantification (LOQ, three replicates <20 % CV within 20 % accuracy) for myostatin, propeptide, GASP-1, and FLRG were 0.01 (0.248), 0.015 (0.42), 0.01 (0.607), and 0.02 nM (0.258 ng), respectively. Standard curves for the proteins are presented in Fig. 1. The intra-assay variability between five replicates of myostatin, propeptide, GASP-1, and FLRG at four different concentrations was less than 12, 10, 8, and 9 %, respectively (Additional file 1: Table S1). The inter-assay variability at different concentrations of myostatin, propeptide, GASP-1, and FLRG was less than 21, 13, 11, and 8 %, respectively (Additional file 2: Table S2). The percent recovery of myostatin, FLRG, and GASP-1 spiked into a pooled serum sample ranged from 65 to 88 % (Additional file 3: Table S3). Collectively, these data validate a specific and sensitive LC-MS/MS method for determination of even very low concentrations of mature and propeptide forms of myostatin and two of its inhibitors in human serum.

### Clinical characteristics of the study subjects

Clinical characteristics of the younger, older, and sarcopenic groups are shown in Table 2, and biochemical parameters are shown in Table 3, stratified by sex. As designed, sarcopenic older women and men had significantly less lean mass (both relative appendicular skeletal muscle mass (ASM) and total body lean mass (TBLM), all *p* < 0.05) and lower grip and knee extension strength than younger and older subjects of the same sex (all *p* < 0.05). Further, younger, older and older sarcopenic men had greater lean mass (both relative ASM and TBLM; all *p* < 0.001) than the corresponding group of women. In fact, the sarcopenic older men not only had greater lean mass than sarcopenic older women but also had greater lean mass than both the younger and older groups of women (Table 1). Consequently, in addition to using *absolute* circulating myostatin concentrations in our analyses, we calculated and used *relative* myostatin and propeptide concentrations by normalizing to TBLM. This facilitated interpretation of myostatin and propeptide concentrations within the context of a given amount of lean mass.

**Table 2:** Descriptive characteristics of younger, older, and sarcopenic older women and men (*n* = 240)

	Younger	Older	Sarcopenic
	(*n* = 40)	(*n* = 40)	(*n* = 40)
Women			
Clinical variables			
Age (years)	32.3 ± 5.5	76.0 ± 8.6*	78.4 ± 8.2**
Weight (kg)	69.2 ± 17.4	78.2 ± 13.9*	68.1 ± 11.9***
Height (cm)	165 ± 6.3	160 ± 6.0*	160 ± 6.5**
BMI (kg/m^2^)	25.3 ± 5.9	30.6 ± 4.6*	26.7 ± 3.9***
Relative ASM (kg/m^2^)	5.9 ± 0.6	6.1 ± 0.7	4.9 ± 0.4**^,^***
TBLM (kg)	37.4 ± 3.9	37.9 ± 5.3	31.1 ± 3.3 **^,^***
TBLM/weight	0.56 ± 0.11	0.49 ± 0.07*	0.47 ± 0.07**
TBFM (kg)	28.2 ± 14.5	37.4 ± 10.4*	33.4 ± 9.7
TBFM/weight	0.38 ± 0.11	0.47 ± 0.07*	0.48 ± 0.07**
Muscle strength			
Grip strength (kg)	27.5 ± 5.1	22.7 ± 5.1*	19.9 ± 3.4*****
Knee extensor strength (kg)	69.6 ± 16.9	52.7 ± 16.5*	42.9 ± 9.8*****
Physical activity			
Energy expenditure (kcal/week)	30837 ± 7744	25399 ± 6125*	22645 ± 6451**
Men			
Clinical variables			
Age (years)	33.0 ± 3.8	74.8 ± 7.3*	78.9 ± 6.8**^,^***
Weight (kg)	92.3 ± 16.7	89.7 ± 15.6	75.9 ± 9.6**^,^***
Height (cm)	179 ± 5.8	175 ± 6.0*	171 ± 7.2**^,^***
BMI (kg/m^2^)	28.8 ± 4.7	29.2 ± 4.0	26.0 ± 2.4**^,^***
Relative ASM (kg/m^2^)	8.5 ± 1.0	7.9 ± 0.4*	6.7 ± 0.5**^,^***
TBLM (kg)	57.6 ± 7.3	54.7 ± 4.2	45.9 ± 4.9**^,^***
TBLM/weight	0.63 ± 0.08	0.62 ± 0.08	0.61 ± 0.07
TBFM (kg)	30.7 ± 12.0	31.6 ± 12.5	26.5 ± 7.3***
TBFM/weight	0.32 ± 0.08	0.34 ± 0.08	0.34 ± 0.06
Muscle strength			
Grip strength (kg)	50.4 ± 9.5	39.2 ± 7.4*	33.7 ± 7.4**^,^***
Knee extensor strength (kg)	122 ± 33.7	80.7 ± 19.1*	61.7 ± 16.8**^,^***
Physical activity			
Energy expenditure (kcal/week)	40136 ± 14350	34222 ± 9090*	27009 ± 7008**^,^***

Values are presented as mean ± SD and *p* values

*BMI* body mass index, *ASM* appendicular skeletal muscle mass, *TBLM* total body lean mass, *TBFM* total body fat mass

*p < 0.05 younger vs. older; **p < 0.05 younger vs. sarcopenic; ***p < 0.05 older vs. sarcopenic

**Table 3:** Biochemical parameters in younger, older and sarcopenic older women and men (n = 240)

	Younger	Older	Sarcopenic
	(*n* = 40)	(*n* = 40)	(*n* = 40)
Women			
Biochemical variables			
Myostatin (ng/mL)	5.5 (3.2–7.3)	7.3 (5.7–11.7)*	5.3 (4.2–8.0)***
Myostatin/TBLM (ng/ml/kg)	0.142 (0.091–0.195)	0.199 (0.138–0.327)*	0.175 (0.145–0.255)**
Propeptide (ng/ml)	7.1 (5.2–10.8)	11.4 (7.3–20.1)*	10.0 (6.5–12.4)***
Propeptide/TBLM (ng/ml/kg)	0.194 (0.139–0.294)	0.292 (0.186–0.203)*	0.307 (0.203–0.417)**
FLRG (ng/mL)	5.1 (4.3–6.0)	7.3 (6.2–9.7)*	8.3 (6.7–10.6)**
GASP1 (ng/mL)	7.6 (6.5–10.1)	9.4 (7.2–11.0)	9.2 (7.1–12.2)**
Total 25-(OH)D (ng/mL)	25.0 (17.3–34.8)	20.0 (14.3–24.0)*	18.5 (14.0–24.8)**
IGF-1 (ng/mL)	235 (191–305)	141 (93–183)*	105 (78–147)**^,^***
IGF-2 (ng/mL)	1160 (958–1375)	1112 (933–1367)	1167 (884–1336)
IGFBP-2 (ng/mL)	406 (190–621)	447 (308–775)	635 (539–987)**^,^***
IGFBP-3 (ng/mL)	4580 (4099–5094)	4096 (3536–4513)*	3913 (3171–4389)**
Total E_1_ (pg/mL)	47.0 (33.3–57.8)	31.0 (19.0–37.0)*	23.0 (16.0–29.0)**
Total E_2_ (pg/mL)	54.0 (30.5–108.5)	5.6 (4.2–9.1)*	4.9 (3.2–7.3)**
Total T (ng/dL)	23.5 (18.0–30.0)	16.0 (12.0–24.0)*	21.0 (11.0–30.0)
Bio E_2_ (pg/mL)	15.0 (8.2–28.2)	2.1 (1.0–3.2)*	1.0 (0.6–1.7)**^,^***
Bio T (ng/dL)	1.5 (0.9–2.1)	1.5 (1.0–2.6)	1.1 (0.7–1.8)***
SHBG (nmol/L)	63.1 (45.0–95.6)	40.5 (23.2–57.8)*	52.8 (46.7–73.0)***
Men			
Biochemical variables			
Myostatin (ng/mL)	10.5 (9.0–14.4)	7.1 (5.4–8.8)*	6.9 (4.4–8.9)**
Myostatin/TBLM (ng/ml/kg)	0.192 (0.156–0.241)	0.131 (0.101–0.164)*	0.153 (0.092–0.192)**
Propeptide (ng/ml)	15.7 (11.7–19.4)	11.8 (7.6–15.7)	10.8 (6.4–14.9)**
Propeptide/TBLM (ng/ml/kg)	0.276 (0.207–0.328)	0.216 (0.137–0.305)	0.240 (0.153–0.309)
FLRG (ng/mL)	4.5 (3.8–5.3)	7.5 (6.1–9.4)*	7.4 (6.3–9.9)**
GASP1 (ng/mL)	9.4 (7.5–10.6)	9.4 (7.3–10.7)	10.3 (8.9–11.9)
Total 25-(OH)D (ng/mL)	23.5 (17.0–31.8)	21.5 (17.0–25.8)	21.0 (16.0–26.8)
IGF-1 (ng/mL)	344 (292–446)	193 (114–245)*	169 (103–245)**
IGF-2 (ng/mL)	1099 (1005–1226)	961 (774–1075)*	964 (785–1071)**
IGFBP-2 (ng/mL)	244 (142–361)	547 (391–728)*	758 (531–1049)**
IGFBP-3 (ng/mL)	4469 (3833–4988)	3202 (2596–3581)*	3230 (2613–4019)**^,^***
Total E_1_ (pg/mL)	32.5 (25.3–38.0)	35.0 (30.0–45.0)*	31.0 (25.5–44.0)
Total E_2_ (pg/mL)	23.0 (19.0–30.0)	25.0 (20.0–28.0)	23.0 (18.0–29.0)
Total T (ng/dL)	462 (392–549)	424 (321–526)	484 (388–609)
Bio E_2_ (pg/mL)	12.8 (11.0–17.5)	9.5 (7.1–11.0)*	7.4 (5.5–10.0)**
Bio T (ng/dL)	133 (115–156)	50.8 (37.0–63.5)*	50.8 (37.4–62.7)**
SHBG (nmol/L)	25.1 (19.4–31.3)	45.5 (39.0–58.6)*	50.3 (39.9–64.0)**

Values are presented as median (IQR)

*TBLM* total body lean mass, *FLRG* follistatin-related gene protein, *GASP-1* growth and serum protein-1, *25(OH)D* 25-hydroxyvitamin D, *IGF* insulin-like growth factor, *IGFBP* IGF-binding protein, *E*_*1*_ estrone, *E*_*2*_ estradiol, *T* testosterone; *SHBG* sex hormone-binding globulin

*p < 0.05 younger vs. older; **p < 0.05 younger vs. sarcopenic; ***p < 0.05 older vs. sarcopenic

### Circulating concentrations of myostatin and myostatin-related proteins in women and men

**Fig. 2:**
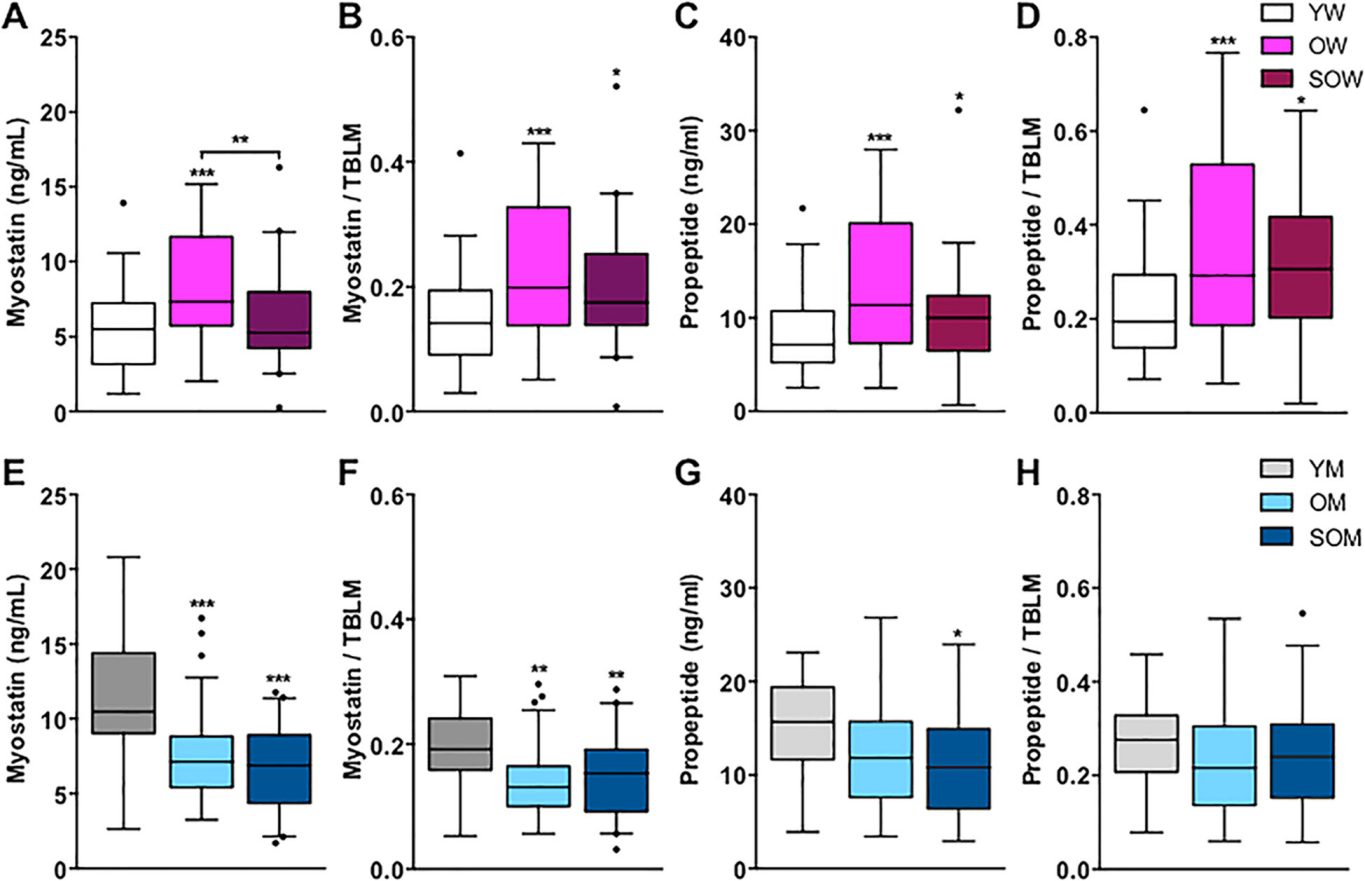
Circulating myostatin and propeptide levels by LC-MS/MS in younger and older women and men. *Box plots* (25–75 percentile) and *whiskers* (Tukey method) comparing serum concentrations of **a** myostatin, **b** myostatin relative to total body lean mass (TBLM), **c** propeptide, and **d** propeptide relative to TBLM between younger women (*YW*), older women (*OW*), and sarcopenic older women (*SOW*). Comparisons of circulating concentrations of absolute and relative concentrations of myostatin and propeptide between corresponding groups of men are also illustrated (**e**-**h**). *, **, and *** denote *p* < 0.05, 0.01, and 0.001, respectively, compared to the younger group except when denoted with a *bracket*

We first compared myostatin, propeptide, FLRG, and GASP-1 within groups of women and men, separately. Compared to younger women, older women had 33 % higher circulating concentrations of myostatin (*p* < 0.001), while sarcopenic older women had comparable levels (Fig. 2a and Table 3). However, for a given amount of TBLM, older women and sarcopenic older women had 40 and 23 % higher relative myostatin concentrations than younger women, respectively (both *p* < 0.01; Fig. 2b). Similar patterns were observed for propeptide concentrations (Table 3), namely, older women had 61 % higher absolute concentrations of propeptide than younger women while both older women and sarcopenic older women had 51 and 58 % higher relative propeptide levels than younger women (Fig. 2c, d). There were no differences in the ratio of myostatin to propeptide between the groups (data not shown (DNS)). In contrast, younger men had significantly higher absolute myostatin concentrations than older men without or with sarcopenia (both *p* < 0.001; Fig. 2e and Table 3). Of note, the age-associated decrease in circulating myostatin observed in men was maintained even after normalizing to TBLM, as younger men had at least 25 % higher relative myostatin levels than both groups of older men (both *p* < 0.01; Fig. 2f). Propeptide levels were also significantly higher in younger men than sarcopenic older men (Fig. 2g), but no differences were observed between groups after normalizing propeptide concentrations to TBLM (Fig. 2h and Table 3). Similar to women, no differences in the ratio of myostatin to propeptide were observed among the groups of men.

**Fig. 3:**
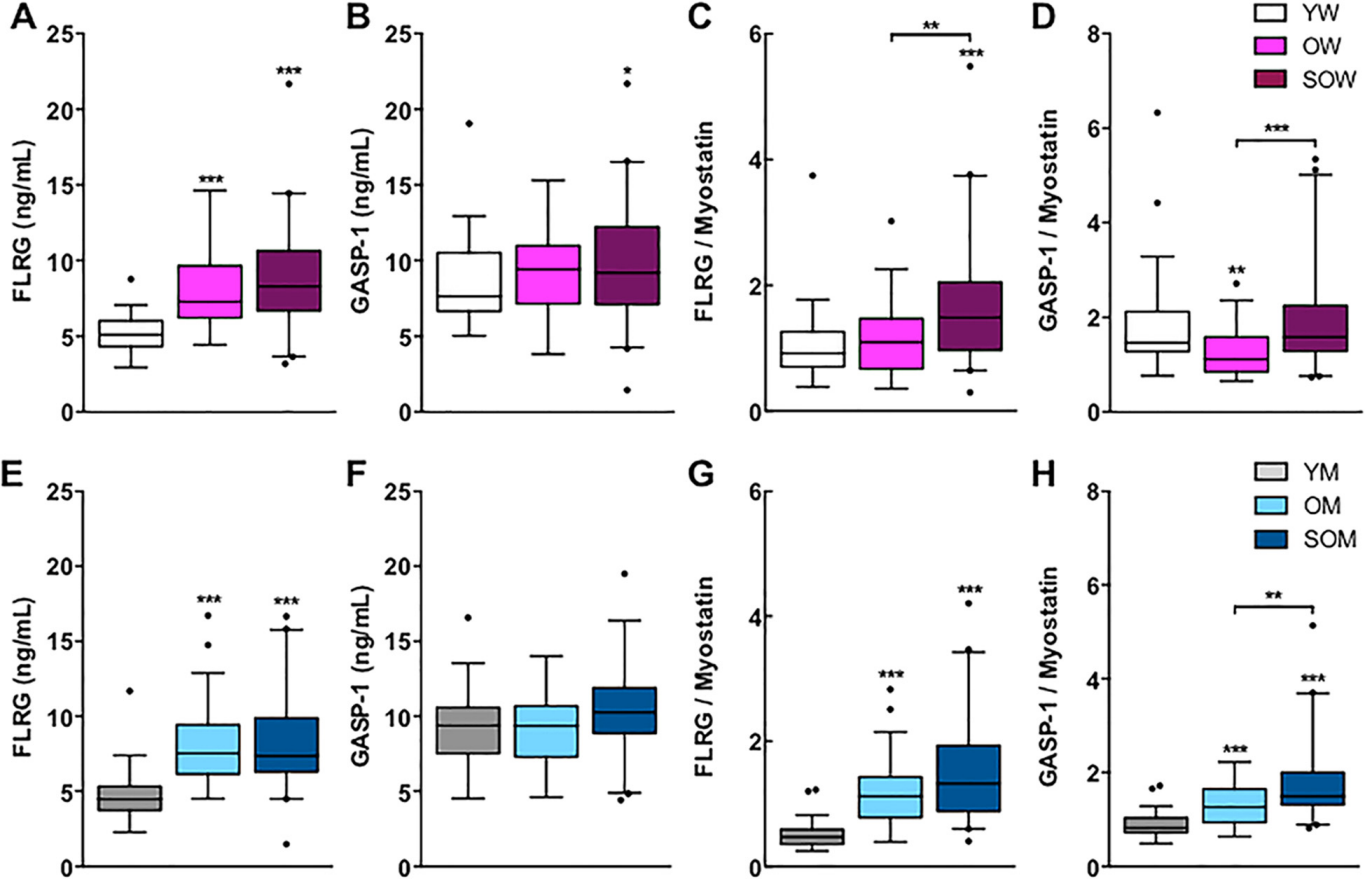
Circulating FLRG and GASP-1 concentrations by LC-MS/MS in younger and older women and men. *Box plots* (25–75 percentile) and *whiskers* (Tukey method) showing comparisons of **a** FLRG, **b** GASP-1, **c** FLRG relative to myostatin, and **d** GASP-1 relative to myostatin between younger women (*YW*), older women (*OW*) and sarcopenic older women (*SOW*). Comparisons of myostatin inhibitors and their ratios to myostatin between corresponding groups of men are also illustrated (**e**-**h**). *, **, and *** denote *p* < 0.05, 0.01, and 0.001, respectively, compared to the younger group except when denoted with a *bracket*

We next explored circulating concentrations of two inhibitors of myostatin, FLRG and GASP-1, in all subjects. Compared to younger women, FLRG levels were 43 and 62 % higher in older women and sarcopenic older women, respectively (both *p* < 0.001; Fig. 3a and Table 3). GASP-1 changed in a like manner, but differences were only significant between younger women and sarcopenic older women (*p* < 0.05; Fig. 3b). We next assessed the ratios of FLRG to myostatin and GASP-1 to myostatin. Sarcopenic older women had the highest ratios of FLRG to myostatin compared to older and younger subjects (Fig. 3c), while a modest decrease in the ratio of GASP-1 to myostatin was observed in older women compared to younger and sarcopenic older women (Fig. 3d). Similar to women, circulating FLRG concentrations were 68 % higher in older men and 64 % higher in sarcopenic older men than in younger men (both *p* < 0.001; Fig. 3e and Table 3), but trends for age-associated increases in GASP-1 were not significant (Fig. 3f). Older men and sarcopenic older men had higher ratios of both FLRG and GASP-1 to myostatin than younger men (all *p* < 0.001; Fig. 3g, h, respectively). In addition, sarcopenic older men had significantly higher ratios of GASP-1 to myostatin than older men (*p* < 0.01).

### Sex differences in circulating concentrations of myostatin and myostatin inhibitors

**Fig. 4:**
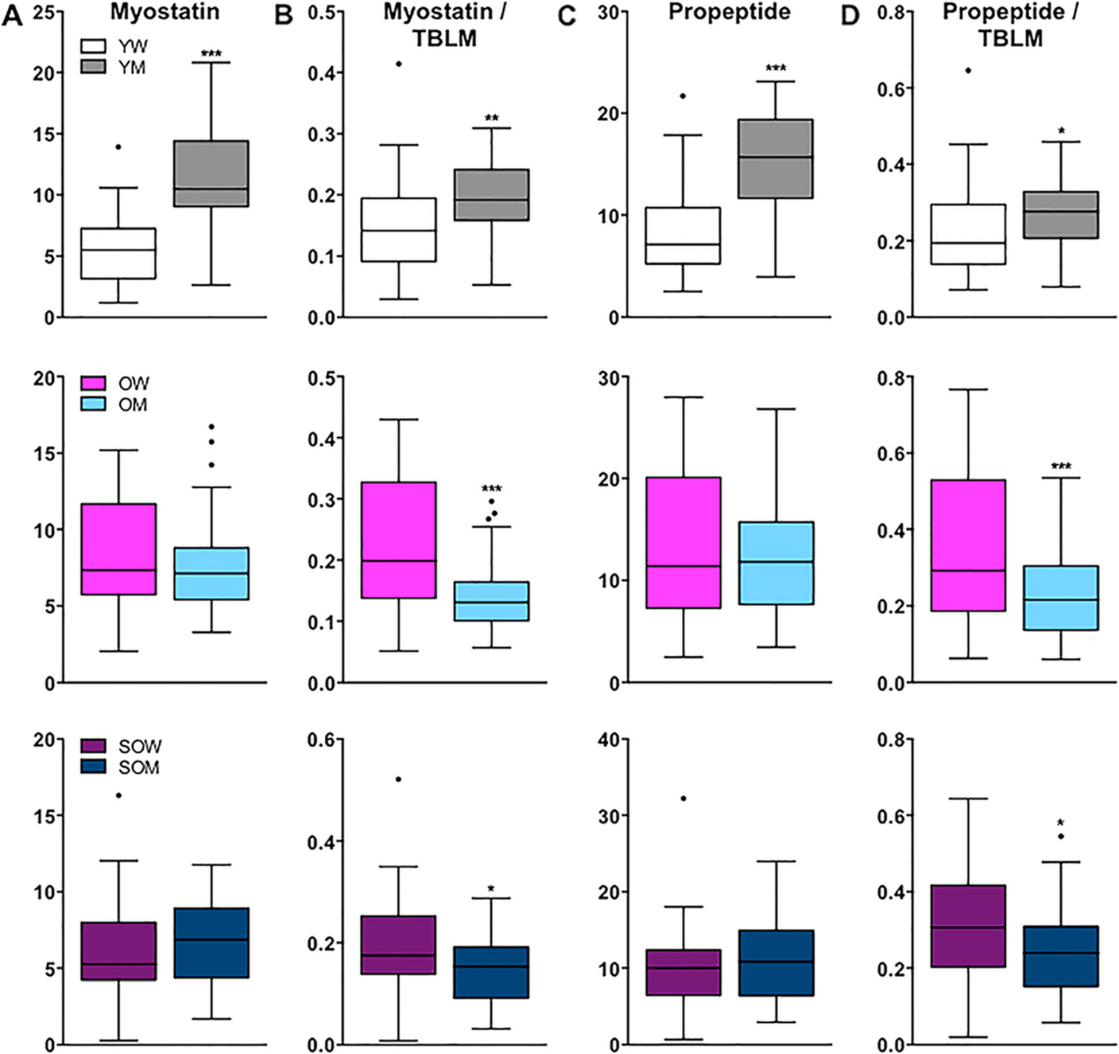
Circulating myostatin and propeptide levels in women compared to men. *Box plots* (25–75 percentile) and *whiskers* (Tukey method) showing comparisons of **a** myostatin, **b** myostatin relative to total body lean mass (TBLM), **c** propeptide, and **d** propeptide relative to TBLM between younger women (*YW*) and younger men (*YM*) (*top panel*), older women (*OW*) and older men (*OM*) (*middle panel*), and sarcopenic OW (*SOW*) and sarcopenic OM (*SOM*) (*bottom panel*). * and *** denote *p* < 0.05 and 0.001, respectively

In separate analyses, we compared myostatin, propeptide, FLRG, and GASP-1 concentrations in women to those in the corresponding group of men. In all subjects combined, circulating myostatin levels were 1.3-fold higher in men than in women (median (interquartile range (IQR)) = 7.8 (6.0–10.5) versus 6.0 (4.3–8.2) ng/mL, *p* < 0.001), even after expressing myostatin relative to TBLM (*p* < 0.01). Similarly, absolute (12.9 (8.6–17.5) versus 9.7 (6.1–13.4)) and relative propeptide concentrations were >1.3-fold higher in men than in women (both *p* < 0.01). Among younger subjects, men had nearly twofold higher myostatin levels as compared to younger women (*p* < 0.001) (Fig. 4a); this difference was diminished after expressing myostatin relative to TBLM (Fig. 4b) but remained significant (*p* < 0.01). Similar observations were made for propeptide, with younger men having greater than twofold higher absolute concentrations (*p* < 0.001; Fig. 4c) and 42 % higher relative concentrations than younger women (*p* < 0.05; Fig. 4d). In older subjects and sarcopenic older subjects, no differences in absolute myostatin or propeptide concentrations were observed between women and men (Fig. 4a and c, respectively). However, after adjusting for differences in TBLM, both older women and sarcopenic older women had 52 and 14 % higher relative myostatin levels than the corresponding groups of men (*p* < 0.001 and 0.05, respectively; Fig. 4b). Older women and sarcopenic older women also had 35 and 28 % higher propeptide levels relative to TBLM than the corresponding groups of men (*p* < 0.01 and 0.05, respectively; Fig. 4d).

**Fig. 5:**
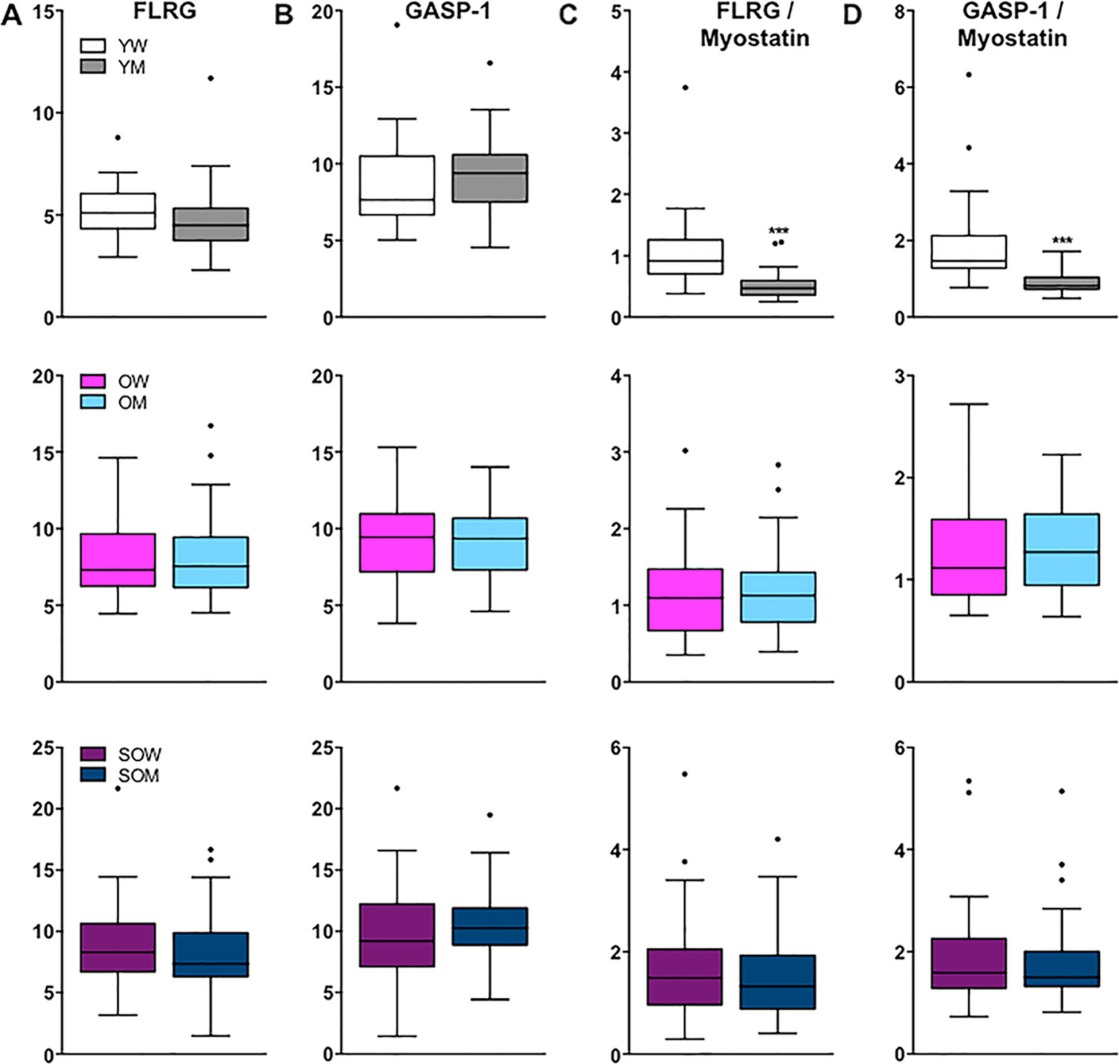
Circulating FLRG and GASP-1 concentrations in women compared to men. *Box plots* (25–75 percentile) and *whiskers* (Tukey method) showing comparisons of **a** FLRG, **b** GASP-1, **c** FLRG relative to myostatin, and **d** GASP-1 relative to myostatin between younger women (*YW*) and younger men (*YM*) (*top panel*), older women (*OW*) and older men (*OM*) (*middle panel*), and sarcopenic OW (*SOW*) and sarcopenic OM (*SOM*) (*bottom panel*). *** denotes *p* < 0.001

Given the differences in myostatin levels between women and men, we next examined whether circulating concentrations of FLRG and GASP-1 differed. In younger, older, and sarcopenic older groups, FLRG and GASP-1 levels were similar between women and men (Fig. 5a, b, respectively). In younger subjects, the ratios of FLRG and GASP-1 to myostatin were significantly higher in younger women as compared to younger men (both *p* < 0.001; Fig. 5c, d, respectively). In older and sarcopenic older subjects, there were no differences between women and men in the ratios of either FLRG or GASP-1 to myostatin.

### Associations between myostatin and body composition, muscle strength, physical activity, and other circulating biochemical parameters

We next examined associations between circulating myostatin levels and body composition, muscle strength, and physical activity using age-adjusted correlations in women and men, separately (Table 4). Positive albeit weak correlations were observed between myostatin concentrations and relative ASM and TBLM in women and relative ASM in men (all *p* < 0.05). Moreover, in men, a negative correlation was observed between myostatin and total body fat mass (*p* < 0.05). Myostatin also exhibited a significant correlation with grip strength (*p* < 0.05) and a positive trend with knee extensor strength (*p* = 0.073) in men but not in women. No correlations were observed between concentrations of myostatin and the amount of self-reported physical activity. Finally, correlations between myostatin and these clinical parameters are provided for the subgroups of younger, older, and older sarcopenic women and men in Additional files 4: Table S4 and 5: Table S5, respectively.

**Table 4:** Age-adjusted Spearman correlations between circulating myostatin levels and body composition, muscle strength, physical activity and biochemical parameters in women and men

Variable	Women (*n* = 120)		Men (*n* = 120)	
	*r*	*p* value	*r*	*p* value
Body composition				
BMI (kg/m^2^)	0.08	0.414	−0.11	0.250
Relative ASM (kg/m^2^)	*0.24*	*0.010*	*0.24*	*0.008*
TBLM (kg)	*0.21*	*0.024*	0.09	0.339
TBLM/weight	−0.03	0.772	*0.26*	*0.005*
TBFM (kg)	0.05	0.585	*−0.20*	*0.026*
TBFM/weight	0.05	0.642	*−0.27*	*0.003*
Muscle strength				
Grip strength (kg)	0.11	0.239	*0.20*	*0.026*
Knee extensor strength (kg)	0.12	0.211	0.17	0.073
Physical activity				
Energy expenditure (kcal/d)	−0.01	0.928	−0.08	0.376
Biochemical parameters				
FLRG (ng/mL)	*0.28*	*0.002*	*0.26*	*0.004*
GASP1 (ng/mL)	*0.53*	*<0.001*	*0.57*	*<0.001*
Total 25-(OH)D (ng/mL)	−0.04	0.701	−0.05	0.575
IGF-1 (ng/mL)	−0.02	0.871	−0.06	0.515
IGF-2 (ng/mL)	−0.17	0.072	−0.09	0.315
IGFBP-2 (ng/mL)	−0.14	0.132	−0.07	0.439
IGFBP-3 (ng/mL)	−0.15	0.115	−0.07	0.464
Total E_1_ (pg/mL)	0.03	0.777	0.09	0.309
Total E_2_ (pg/mL)	0.02	0.843	0.03	0.778
Total T (ng/dL)	*−0.20*	*0.027*	0.18	0.056
Bioavailable E_2_ (pg/mL)	0.11	0.272	0.13	0.158
Bioavailable T (ng/dL)	0.12	0.188	*0.32*	*<0.001*
SHBG (nmol/L)	*−0.29*	*0.001*	−0.13	0.170

Values are presented as Spearman correlation coefficients (r) and *p* values

*BMI* body mass index, *ASM* appendicular skeletal muscle mass, *TBLM* total body lean mass, *TBFM* total body fat mass, *FLRG* follistatin-related gene protein, *GASP-1* growth and serum protein-1, *25(OH)D* 25-hydroxyvitamin D, *IGF* insulin-like growth factor, *IGFBP* insulin-like growth factor binding protein, *E*_*1*_ estrone, *E*_*2*_ estradiol, *T* testosterone, *SHBG* sex hormone-binding globulin

In women and men, myostatin levels exhibited modest age-adjusted correlations with FLRG (both *p* < 0.01) and stronger correlations with GASP-1 (both *p* < 0.001). Analysis of other circulating biochemical parameters revealed a negative correlation between myostatin and total testosterone and sex hormone-binding globulin in women (*p* < 0.05 and 0.001, respectively). In men, myostatin had a positive association with bioavailable testosterone (*p* < 0.001, respectively). No correlations were observed between myostatin and vitamin D or circulating components of the insulin-like growth factor (IGF) system, including IGF-1, IGF-2, IGF-binding protein (BP)-2, and IGFBP-3. Correlations between myostatin and the biochemical parameters are provided for the subgroups of younger, older, and older sarcopenic women and men in Additional files 4: Table S4 and 5: Table S5, respectively.

## Discussion

In this study, we developed a highly specific and accurate multiplexed LC-MS/MS assay for measuring circulating concentrations of mature and propeptide forms of the muscle-derived protein, myostatin, and two of its inhibitors, FLRG and GASP-1, in human serum. Using this novel approach and a well-characterized population-based sample, we show that absolute and relative concentrations of myostatin and propeptide are higher in younger men than younger women, increase with age in women, but in fact decrease with age in men. Intriguingly, these age-associated changes result in much higher circulating myostatin and propeptide concentrations per unit of lean mass in older women than older men. We also demonstrate that circulating concentrations of FLRG and, to a lesser extent, GASP-1 increase similarly in women and men with age, particularly in the context of sarcopenia. Finally, we report that circulating concentrations of myostatin exhibit positive, but not robust, age-adjusted correlations with relative ASM in both sexes.

As highlighted here and previously [31, 32], there are several challenges to the specific and accurate measurement of circulating myostatin concentrations using traditional antibody-based approaches, such as RIA, ELISA, and Western blotting. Through chromatographic separation and the use of peptide sequences, or “fingerprints,” that are unique to and, in particular, distinct from GDF-11, LC-MS/MS provides a highly specific method to quantify myostatin. Using this approach, we observed myostatin levels of 8.6 ± 3.7 ng/ml in men and 6.7 ± 3.3 ng/ml in women. These concentrations are considerably lower than the mean values (26.7 to >100 ng/ml) reported in several recent studies of healthy adults using commercial ELISA kits [16, 18, 19, 33–35] but similar to the results obtained in healthy adults using an ELISA comprised of proprietary and presumably more specific, reagents ([15, 17]. In addition to improved specificity, LC-MS/MS has better sensitivity for quantifying low abundance proteins than antibody-based approaches. By coupling immunopurification and LC-MS/MS, we observed both a LOD and a LOQ of 0.01 nM, or 0.248 ng, for myostatin. In comparison, Peiris et al. observed that the LOD for recombinant myostatin proteins by Western blot was ~83.33 nM, or 2000 ng [32]. We established similarly low LOD and LOQ values for propeptide, FLRG, and GASP-1. Therefore, this novel multiplexed LC-MS/MS assay offers a highly specific and sensitive means to quantify circulating concentrations of myostatin and myostatin-related proteins in a single small (400 ul) sample of human serum.

Myostatin is a promising therapeutic target to improve muscle health [36]. Several pharmacological approaches have been developed, including neutralizing antibodies, propeptides, soluble decoy receptors, and receptor antagonists. Such interventions have been shown to increase skeletal muscle mass and improve parameters of strength and physical function in preclinical models of aging and disease [12, 37, 38]. A number of early phase clinical trials are underway [36, 39]. It is therefore surprising then, that relatively little is known about the relationship between circulating myostatin concentrations and skeletal muscle mass in human conditions associated with its loss or degeneration. Indeed such data could inform the selection of indications or individuals that may be most responsive to targeted interventions. In the present study, we demonstrate contrasting age-associated changes in myostatin and propeptide levels in women and men. Specifically, we observed higher absolute and relative circulating concentrations in older women compared to younger women and lower concentrations in older men compared to younger men. Unexpectedly, we also measured higher concentrations of myostatin and propeptide per unit of lean mass in older women and sarcopenic older women than in corresponding groups of men. The prevalence of sarcopenia is higher in women than men; however, we can only speculate that myostatin plays a causal role in age-associated muscle loss in women and that women may be more responsive than men to anti-myostatin therapies. In both sexes, we failed to see meaningful differences in myostatin concentrations or the ratio of myostatin to propeptide between older subjects and sarcopenic older subjects. Of note, we studied healthy older persons without chronic diseases associated with the deterioration of skeletal muscle, including cancer, chronic heart failure, chronic obstructive pulmonary disease, diabetes, chronic kidney disease, and human immunodeficiency virus. Future research is needed to determine the extent to which myostatin concentrations, or the ratio of myostatin to propeptide, are associated with skeletal muscle mass and function in the context of such conditions.

In 1962, Bullough and Lawrence first proposed that “diffusible substances,” or chalones, regulate the mass of the specific tissue from which they were derived [1, 40]. Shortly after its discovery as a protein synthesized and secreted by skeletal muscle, Lee and McPherron highlighted the potential for myostatin to be a muscle chalone [41]. As a chalone, myostatin may be an evolutionarily conserved mechanism that was selected to prevent the allocation of limited resources to the further development and maintenance of the tissue from which it is derived. In younger women, this may have been critical for reproduction. Consistent with the concept of *antagonistic pleiotropy*, this early life benefit of myostatin may have later life costs, namely, the excessive deterioration of skeletal muscle. With the significant extension in life expectancy beyond the menopause, it is plausible that counter regulatory mechanisms could not be selected for and, consequently, myostatin contributes to the age-associated loss of skeletal muscle in women. In men on the other hand, myostatin is highest in younger men with the greatest muscle mass and, as would be anticipated for a chalone, lowest in older men with the least muscle mass. This same pattern is observed for sclerostin, which is synthesized in and secreted by osteocytes. Sclerostin functions as a potent negative regulator of the Wnt/β-catenin signaling pathway to inhibit bone formation [42]. Serum sclerostin levels are positively associated with total body bone mass in both women and men [43]. The reason for sexually dimorphic age-related changes in myostatin is unclear. We observed that FLRG and GASP-1 are higher in younger women than younger men and increase with age in both sexes and even more so in those who are sarcopenic. This is the first report of FLRG and GASP-1 serum concentrations in women; however, in a smaller study using ELISAs, Ratvekius et al. observed no differences or trends for a decrease in FLRG and GASP-1 in older men compared to younger men. Furthermore, our data fail to show meaningful relationships between circulating myostatin concentrations in women and established mediators of skeletal muscle mass, including bioavailable testosterone, IGF-1, and IGF-2. In men, we observed positive, not negative, associations between circulating myostatin and bioavailable estrogen and testosterone concentrations. Additional research is needed to further understand what factors regulate myostatin abundance and/or activity and how such factors are affected by aging in both women and men.

Our study provides important insights into age-related changes and sex differences in the circulating concentrations of myostatin and its related proteins in healthy adults. However, it is important to recognize the cross-sectional design of our study. Longitudinal studies are needed to better define the relationships between myostatin, propeptide, FLRG and GASP-1 and age- and disease-related changes in skeletal muscle mass and performance, and the utility of these proteins as a biomarkers of current and future muscle health. Our sample was also predominantly white, and underrepresented with respect to persons of African, Asian, Hispanic, Latino, American Indian, and Alaskan Native ancestry groups. To our knowledge, the influence of origin on myostatin concentrations has not been investigated. Furthermore, our assay has notable strengths, including the ability to precisely monitor four analytes in a single sample of merely 400 ul of serum. However, while we can specifically monitor the abundance of C-terminal (mature) and N-terminal (propeptide) regions unique to myostatin, at this time, we are not able to define the stoichiometry of free (active) versus bound (latent) forms in vivo*.* Of note, we did attempt an acid activation step in pooled serum to overcome this hurdle; however, we had reduced recovery of all proteins with the exception of propeptide, which did not change. We therefore chose to immunoprecipitate under physiological conditions without acid activation. Even so, we believe this multiplexed LC-MS/MS approach represents the current upper limit of specificity and sensitivity for assessing myostatin, propeptide, FLRG, and GASP-1 in human clinical samples, and that our study represents the most comprehensive assessment of these proteins in both women and men to date.

## Conclusions

We have developed a highly specific and sensitive LC-MS/MS-based method for measuring concentrations of myostatin, propeptide, FLRG, and GASP-1 in a single small volume of human serum. We propose that (1) the age-associated increase in myostatin levels in women may contribute to their lower muscle mass and higher prevalence of sarcopenia relative to men; (2) myostatin acts as a homeostatic regulator of muscle mass in men, that is, the age-related loss of muscle in men is coupled with a decrease in myostatin and an increase in its inhibitors; (3) FLRG and GASP-1 increase with age and in the context of sarcopenia to inhibit the catabolic actions of myostatin; and (4) circulating concentrations of myostatin provide a significant, albeit weak biomarker of muscle mass in relatively healthy adult women and men. This novel method will enable future studies to determine the extent to which circulating concentrations of myostatin and its inhibitors change in the context of conditions associated with muscle loss or degeneration and, potentially, help identify individuals and conditions that will best respond to therapies that block myostatin signaling.

## Additional files

Additional file 1: Table S1. Intra-assay precision of five replicate measures of recombinant myostatin, propeptide FLRG, and GASP-1 at four concentrations diluted in 5 % bovine serum albumin in phosphate buffered saline. A pooled human serum sample was also analyzed. Additional file 2: Table S2. Inter-assay variability for immunoaffinity purification and LC-MS/MS measures of recombinant myostatin, propeptide, FLRG, and GASP-1. Proteins were diluted together at the indicated concentrations in 5 % bovine serum albumin in phosphate buffered saline. Measures were performed five times over a period of 21 days. A pooled human serum sample was analyzed similarly. Additional file 3: Table S3. Percent recovery of myostatin, FLRG, and GASP-1. A human serum pool was spiked (+) at two concentrations (CONC) of the given analyte, and the percent recovery was determined relative to the calculated value of the endogenous level plus the spiked protein. Additional file 4: Table S4. Spearman correlations between circulating myostatin levels and body composition, muscle strength, physical activity, and biochemical parameters in women. Additional file 5: Table S5. Spearman correlations between circulating myostatin levels and body composition, muscle strength, physical activity, and biochemical parameters in men.
