# Supplementary material for: Myostatin as a mediator of sarcopenia versus homeostatic regulator of muscle mass: insights using a new mass spectrometry-based assay
Source: Skelet Muscle. 2015 Jul 15;5:21. doi: 10.1186/s13395-015-0047-5 (PMC4502935; doi:10.1186/s13395-015-0047-5)
Supplement: Additional file 1: Table S1. — Intra-assay precision of five replicate measures of recombinant myostatin, propeptide FLRG, and GASP-1 at four concentrations diluted in 5 % bovine serum albumin in phosphate buffered saline. A pooled human serum sample was also analyzed. [file 13395_2015_47_MOESM1_ESM.doc]

**Supplemental Table 1.** Intra-assay precision of five replicate measures of recombinant myostatin, propeptide FLRG and GASP-1 at four concentrations diluted in 5% bovine serum albumin in phosphate buffered saline. A pooled human serum sample was also analyzed.

|  | **Myostatin** | **Propeptide** | **FLRG** | **GASP-1** |
| --- | --- | --- | --- | --- |
| ***0.03nM*** | | | | |
| Mean | 0.03 | 0.04 | 0.04 | 0.03 |
| SD | 0.00 | 0.00 | 0.00 | 0.00 |
| CV | 1.46 | 5.21 | 7.75 | 1.75 |
| ***0.19nM*** | | | | |
| Mean | 0.19 | 0.19 | 0.24 | 0.20 |
| SD | 0.01 | 0.01 | 0.01 | 0.00 |
| CV | 6.42 | 7.76 | 4.74 | 1.20 |
| ***0.38nM*** | | | | |
| Mean | 0.45 | 0.43 | 0.36 | 0.42 |
| SD | 0.05 | 0.04 | 0.02 | 0.02 |
| CV | 11.49 | 9.82 | 6.57 | 5.09 |
| ***0.75nM*** | | | | |
| Mean | 0.85 | 0.74 | 0.73 | 0.80 |
| SD | 0.04 | 0.05 | 0.03 | 0.07 |
| CV | 4.50 | 7.17 | 3.69 | 8.57 |
| ***Pooled Human Serum*** | | | | |
| Mean | 0.24 | 0.22 | 0.20 | 0.09 |
| SD | 0.01 | 0.02 | 0.01 | 0.00 |
| CV | 4.89 | 8.04 | 5.96 | 3.46 |
