# Supplementary material for: Myostatin as a mediator of sarcopenia versus homeostatic regulator of muscle mass: insights using a new mass spectrometry-based assay
Source: Skelet Muscle. 2015 Jul 15;5:21. doi: 10.1186/s13395-015-0047-5 (PMC4502935; doi:10.1186/s13395-015-0047-5)
Supplement: Additional file 2: Table S2. — Inter-assay variability for immunoaffinity purification and LC-MS/MS measures of recombinant myostatin, propeptide, FLRG, and GASP-1. Proteins were diluted together at the indicated concentrations in 5 % bovine serum albumin in phosphate buffered saline. Measures were performed five times over a period of 21 days. A pooled human serum sample was analyzed similarly. [file 13395_2015_47_MOESM2_ESM.doc]

**Supplemental Table 2.** Inter-assay variability for immunoaffinity purification and LC-MS/MS measures of recombinant myostatin, propeptide, FLRG and GASP-1. Proteins were diluted together at the indicated concentrations in 5% bovine serum albumin in phosphate buffered saline. Measures were performed five times over a period of twenty-one days. A pooled human serum sample was analyzed similarly.

|  | **Myostatin** | **Propeptide** | **FLRG** | **GASP-1** |
| --- | --- | --- | --- | --- |
| ***0.03nM*** | | | | |
| Mean | 0.04* | 0.03 | 0.03 | 0.03 |
| SD | 0.01* | 0.00 | 0.00 | 0.00 |
| CV | 20.89* | 12.52 | 13.73 | 5.57 |
| ***0.19nM*** | | | | |
| Mean | 0.20 | 0.19 | 0.20 | 0.19 |
| SD | 0.01 | 0.02 | 0.02 | 0.01 |
| CV | 4.26 | 8.75 | 7.79 | 3.28 |
| ***0.38nM*** | | | | |
| Mean | 0.36 | 0.40 | 0.41 | 0.40 |
| SD | 0.03 | 0.03 | 0.04 | 0.03 |
| CV | 7.37 | 6.96 | 10.19 | 7.40 |
| ***Pooled Human Serum*** | | | | |
| Mean | 0.36 | 0.40 | 0.41 | 0.40 |
| SD | 0.03 | 0.03 | 0.04 | 0.03 |
| CV | 7.37 | 6.96 | 10.19 | 7.40 |

*****The myostatin 0.03nM values represent three time points over 21 days. Two of the time points failed due to a loss of instrument sensitivity during acquisition.
