# Supplementary material for: Myostatin as a mediator of sarcopenia versus homeostatic regulator of muscle mass: insights using a new mass spectrometry-based assay
Source: Skelet Muscle. 2015 Jul 15;5:21. doi: 10.1186/s13395-015-0047-5 (PMC4502935; doi:10.1186/s13395-015-0047-5)
Supplement: Additional file 3: Table S3. — Percent recovery of myostatin, FLRG, and GASP-1. A human serum pool was spiked (+) at two concentrations (CONC) of the given analyte, and the percent recovery was determined relative to the calculated value of the endogenous level plus the spiked protein. [file 13395_2015_47_MOESM3_ESM.doc]

**Supplemental Table 3.** Percent recovery of myostatin, FLRG and GASP-1. A human serum pool was spiked (+) at two concentrations (CONC) of the given analyte and the percent recovery was determined relative to the calculated value of the endogenous level plus the spiked protein.

|  | **Myostatin** | | **FLRG** | | **GASP-1** | |
| --- | --- | --- | --- | --- | --- | --- |
|  | CONC  (nM) | Recovery (%) | CONC  (nM) | Recovery (%) | CONC  (nM) | Recovery (%) |
| ***Neat Serum*** | 0.326 | - | 0.132 | - | 0.091 | - |
| ***+ 0.1 nM*** | 0.406 | 80% | 0.220 | 88% | 0.173 | 82% |
| ***+ 0.25 nM*** | 0.521 | 78% | 0.339 | 83% | 0.254 | 65% |
