# Supplementary material for: Myostatin as a mediator of sarcopenia versus homeostatic regulator of muscle mass: insights using a new mass spectrometry-based assay
Source: Skelet Muscle. 2015 Jul 15;5:21. doi: 10.1186/s13395-015-0047-5 (PMC4502935; doi:10.1186/s13395-015-0047-5)
Supplement: Additional file 4: Table S4. — Spearman correlations between circulating myostatin levels and body composition, muscle strength, physical activity, and biochemical parameters in women. [file 13395_2015_47_MOESM4_ESM.doc]

**Supplemental Table 4.** Spearman correlations between circulating myostatin levels and body composition, muscle strength, physical activity and biochemical parameters in women.

| ***Variable*** | ***Younger Women*** | |  | ***Older Women*** | |  | ***Sarcopenic Women*** | |
| --- | --- | --- | --- | --- | --- | --- | --- | --- |
|  | **r** | ***P*** |  | **r** | ***P*** |  | **r** | ***P*** |
| ***Body Composition*** |  |  |  |  |  |  |  |  |
| BMI (kg/m^2^) | 0.04 | 0.828 |  | -0.14 | 0.384 |  | 0.01 | 0.970 |
| Relative ASM (kg/m^2^) | 0.30 | 0.060 |  | -0.04 | 0.791 |  | -0.10 | 0.548 |
| TBLM (kg) | **0.34** | **0.033** |  | -0.16 | 0.318 |  | 0.02 | 0.898 |
| TBLM / Weight | 0.06 | 0.720 |  | 0.04 | 0.788 |  | -0.02 | 0.896 |
| TBFM (kg) | 0.00 | 0.996 |  | -0.09 | 0.564 |  | 0.05 | 0.757 |
| TBFM / Weight | -0.09 | 0.595 |  | -0.04 | 0.785 |  | 0.07 | 0.665 |
| ***Muscle Strength*** |  |  |  |  |  |  |  |  |
| Grip strength (kg) | 0.26 | 0.100 |  | 0.07 | 0.650 |  | -0.22 | 0.165 |
| Knee extensor strength (kg) | 0.10 | 0.547 |  | 0.13 | 0.425 |  | 0.05 | 0.756 |
| ***Physical Activity*** |  |  |  |  |  |  |  |  |
| Energy expenditure (kcal/d) | -0.05 | 0.777 |  | -0.04 | 0.831 |  | 0.01 | 0.935 |
| ***Biochemical Parameters*** |  |  |  |  |  |  |  |  |
| Total 25-(OH)D (ng/mL) | 0.05 | 0.768 |  | 0.05 | 0.764 |  | 0.01 | 0.938 |
| IGF-1 (ng/mL) | -0.26 | 0.099 |  | 0.06 | 0.728 |  | 0.15 | 0.342 |
| IGF-2 (ng/mL) | -0.11 | 0.494 |  | -0.02 | 0.885 |  | -0.26 | 0.101 |
| IGFBP-2 (ng/mL) | 0.20 | 0.224 |  | **-0.35** | **0.027** |  | 0.08 | 0.611 |
| IGFBP-3 (ng/mL) | -0.21 | 0.202 |  | 0.00 | 0.984 |  | -0.13 | 0.430 |
| Total E_2_ (pg/mL) | -0.10 | 0.540 |  | -0.06 | 0.724 |  | **0.33** | **0.042** |
| Total T (ng/dL) | **-0.36** | **0.024** |  | -0.24 | 0.133 |  | 0.06 | 0.700 |
| Bioavailable E_2_ (pg/mL) | -0.21 | 0.369 |  | 0.08 | 0.611 |  | 0.26 | 0.109 |
| Bioavailable T (ng/dL) | 0.17 | 0.289 |  | 0.00 | 0.996 |  | 0.05 | 0.773 |
| SHBG (nmol/L) | **-0.37** | **0.018** |  | -0.27 | 0.095 |  | 0.12 | 0.463 |
| Values are presented as Spearman correlation coefficients (r) and *P*-values. BMI = body mass index; ASM = appendicular skeletal muscle mass; TBLM = total body lean mass; TBFM = total body fat mass; 25(OH)D = 25-hydroxyvitamin D; IGF = insulin-like growth factor; IGFBP = insulin-like growth factor binding protein; E_2_ = estradiol; T = testosterone; SHBG = sex hormone-binding globulin. | | | | | | | | |
