# Supplementary material for: Myostatin as a mediator of sarcopenia versus homeostatic regulator of muscle mass: insights using a new mass spectrometry-based assay
Source: Skelet Muscle. 2015 Jul 15;5:21. doi: 10.1186/s13395-015-0047-5 (PMC4502935; doi:10.1186/s13395-015-0047-5)
Supplement: Additional file 5: Table S5. — Spearman correlations between circulating myostatin levels and body composition, muscle strength, physical activity, and biochemical parameters in men. [file 13395_2015_47_MOESM5_ESM.doc]

**Supplemental Table 5.** Spearman correlations between circulating myostatin levels and body composition, muscle strength, physical activity and biochemical parameters in men.

| ***Variable*** | ***Younger Men*** | |  | ***Older Men*** | |  | ***Sarcopenic Men*** | |
| --- | --- | --- | --- | --- | --- | --- | --- | --- |
|  | **r** | ***P*** |  | **r** | ***P*** |  | **r** | ***P*** |
| ***Body Composition*** |  |  |  |  |  |  |  |  |
| BMI (kg/m^2^) | 0.15 | 0.346 |  | -0.31 | 0.050 |  | -0.19 | 0.232 |
| Relative ASM (kg/m^2^) | **0.42** | **0.007** |  | 0.21 | 0.202 |  | 0.04 | 0.809 |
| TBLM (kg) | **0.36** | **0.021** |  | -0.11 | 0.508 |  | -0.05 | 0.745 |
| TBLM / Weight | 0.14 | 0.400 |  | **0.34** | **0.034** |  | 0.16 | 0.311 |
| TBFM (kg) | -0.05 | 0.771 |  | **-0.33** | **0.036** |  | -0.26 | 0.099 |
| TBFM / Weight | -0.13 | 0.418 |  | **-0.34** | **0.032** |  | -0.27 | 0.097 |
| ***Muscle Strength*** |  |  |  |  |  |  |  |  |
| Grip strength (kg) | **0.39** | **0.013** |  | -0.03 | 0.862 |  | 0.02 | 0.905 |
| Knee extensor strength (kg) | **0.39** | **0.013** |  | -0.02 | 0.911 |  | 0.17 | 0.285 |
| ***Physical Activity*** |  |  |  |  |  |  |  |  |
| Energy expenditure (kcal/d) | 0.10 | 0.529 |  | -0.23 | 0.158 |  | -0.03 | 0.877 |
| ***Biochemical Parameters*** |  |  |  |  |  |  |  |  |
| Total 25-(OH)D (ng/mL) | 0.08 | 0.605 |  | **-0.36** | **0.022** |  | 0.06 | 0.714 |
| IGF-1 (ng/mL) | 0.00 | 0.985 |  | -0.06 | 0.699 |  | -0.14 | 0.375 |
| IGF-2 (ng/mL) | 0.11 | 0.507 |  | **-0.32** | **0.047** |  | -0.14 | 0.384 |
| IGFBP-2 (ng/mL) | 0.04 | 0.813 |  | 0.00 | 0.984 |  | -0.12 | 0.450 |
| IGFBP-3 (ng/mL) | 0.07 | 0.667 |  | 0.12 | 0.457 |  | -0.28 | 0.078 |
| Total E_2_ (pg/mL) | 0.05 | 0.778 |  | 0.08 | 0.641 |  | 0.20 | 0.225 |
| Total T (ng/dL) | 0.10 | 0.549 |  | 0.10 | 0.552 |  | **0.39** | **0.014** |
| Bioavailable E_2_ (pg/mL) | 0.02 | 0.897 |  | 0.19 | 0.254 |  | 0.12 | 0.462 |
| Bioavailable T (ng/dL) | 0.09 | 0.592 |  | 0.17 | 0.311 |  | **0.39** | **0.014** |
| SHBG (nmol/L) | 0.04 | 0.807 |  | -0.19 | 0.246 |  | -0.01 | 0.951 |
| Values are presented as Spearman correlation coefficients (r) and *P*-values. BMI = body mass index; ASM = appendicular skeletal muscle mass; TBLM = total body lean mass; TBFM = total body fat mass; 25(OH)D = 25-hydroxyvitamin D; IGF = insulin-like growth factor; IGFBP = insulin-like growth factor binding protein; E_2_ = estradiol; T = testosterone; SHBG = sex hormone-binding globulin. | | | | | | | | |
